# Lives saved and financial risk protectionfrom scaling up kangaroo mother care in India: an extended cost -effectivenessanalysis

**DOI:** 10.1136/bmjgh-2025-021719

**Published:** 2026-09-02

**Authors:** Tarun Shankar Choudhary, Sarmila Mazumder, Oystein A Haaland, Sunita Taneja, Halvor Sommerfelt, Ole F Norheim, Nita Bhandari, Kjell Arne Johansson

**Affiliations:** 1 Department of Global Public Health and Primary Care, Centre for Intervention Science in Maternal and Child Health, University of Bergen, Bergen, Norway; 2 Department of Global Public Health and Primary Care, Bergen Centre for Ethics and Priority Setting in Health, University of Bergen, Bergen, Norway; 3 Society for Applied Studies, New Delhi, Delhi, India; 4 Department of Global Public Health and Primary Care, University of Bergen, Bergen, Norway; 5 Department of Global Health and Population, Harvard T H Chan School of Public Health, Boston, Massachusetts, USA; 6 Centre for Health Research and Development, Society for Applied Studies, New Delhi, Delhi, India

**Keywords:** India, Health economics, Public Health

## Abstract

**Introduction:**

Kangaroo mother care (KMC) is a proven, low-cost intervention to reduce neonatal mortality, yet its coverage remains limited in most low- and middle-income countries (LMICs), including India. In this study, we examined the impact of scaling up KMC in India on distribution of health and financial outcomes using an extended cost-effectiveness analysis (ECEA) framework.

**Methods:**

Building on primary data from a randomised controlled trial (RCT), we modelled the health and financial outcomes of scaling up KMC to 95% coverage nationally. We estimated lives saved, disability-adjusted life-years (DALYs) averted, out-of-pocket expenditure (OOPE) averted and cases of catastrophic healthcare expenditure prevented. Estimates were disaggregated by socioeconomic quintiles and geography. We used deterministic and probabilistic sensitivity analyses to assess the robustness of our findings.

**Results:**

Scaling up KMC in India could save around 26 000 neonatal lives annually, avert approximately 1.6 million DALYs, reduce OOPE by around US$13 million and prevent an estimated 57 000 households from experiencing catastrophic health expenditures. Three-fourths of the benefits would be concentrated in the lowest two socioeconomic quintiles. Six high-burden states, including Uttar Pradesh, Bihar and Madhya Pradesh, accounted for 90% of the health and financial gains. The incremental cost-effectiveness ratio ranged from 0.08 to 0.17 times gross domestic product per capita for India across different quintiles. Probabilistic sensitivity analysis showed scaling up KMC would be cost-effective in 97.8% of simulations.

**Conclusion:**

Our findings suggest that scaling up KMC is cost-effective and can reduce health inequities, particularly benefiting the lowest socioeconomic groups and high-burden states. Incorporating geographical disaggregation and using primary RCT data into ECEA enhance its utility for informing evidence-based, equity-focused health policies in LMICs.

**WHAT IS ALREADY KNOWN ON THIS TOPIC**

- Kangaroo mother care (KMC) is proven to reduce neonatal mortality among preterm and low-birthweight infants, but extended cost-effectiveness analyses (ECEAs) of KMC are lacking in India and other low-income countries.
- Previous ECEAs in maternal and child health mostly relied on secondary data, focused narrowly on socioeconomic equity and seldom examined geographical disparities that are critical in settings with a decentralised health system.

**WHAT THIS STUDY ADDS**

- Using primary data from a randomised controlled trial (RCT), we provide robust estimates of KMC’s health, equity and financial protection impacts in India.
- By introducing geographical disaggregation alongside socioeconomic analysis, we show that three-fourths of the benefits accrue to the poorest households, and six high-burden states account for 90% of the health and financial gains.

**HOW THIS STUDY MIGHT AFFECT RESEARCH, PRACTICE OR POLICY**

- Our findings demonstrate that scaling up KMC through universal public finance is a cost-effective and equity-enhancing intervention in India.
- They highlight the value of ECEA in extending the policy relevance of RCTs and strengthen the case for prioritising equity-focused maternal and child health strategies in low- and middle-income countries.

## Introduction

 In India, half of healthcare is financed out-of-pocket expenditure (OOPE), and around 16% of households experience catastrophic payments due to healthcare seeking.^1^ High levels of OOPE impose substantial financial burden and constitute a significant barrier to healthcare access, disproportionately affecting the most vulnerable households. This contributes to an increased risk of impoverishment, delayed care seeking—often from informal sources—and, in extreme cases, no care seeking.^2–4^ In 2017–2018, 15% of the Indian households who accessed healthcare were pushed below the poverty line because of OOPE.^1^ Reducing financial vulnerability and protection from health-related financial hardship is critical for attainment of the Sustainable Development Goals 1 and 3 and universal health coverage.^5 6^ Therefore, the decision-making process for introduction of interventions into global and national policies and programmes should include evidence of total effectiveness and incorporate the impact on financial risk protection (FRP) and distributional impact of benefits.

Low birth weight (LBW), defined as birth weight less than 2.5 kg, affects approximately 4 million newborns annually in India. LBW prevalence varies considerably across Indian states, ranging from 3% to 21%.^7^ LBW infants experience substantially higher mortality and morbidity in early life compared with those with normal birth weight and have an increased risk of developing non-communicable diseases later in life.^8^ Furthermore, LBW can impair growth and cognitive development.^8^ Despite progress, India is currently not on track to meet the 2030 targets for reducing LBW prevalence.

Kangaroo mother care (KMC) consists of exclusive breastfeeding and keeping newborns in skin-to-skin contact with their mother or a surrogate as long as possible.^9 10^ The intervention can be initiated in a healthcare facility or at home. KMC reduces the risk of neonatal death by 30% and may improve growth outcomes.^11 12^ While the WHO recommends KMC for all preterm or LBW children, uptake and scale-up remain slow, especially in low- and middle-income countries (LMICs), where the majority of the LBW births occur and the impact of potential KMC scale-up would be largest.^13^

Health and financial benefits of interventions are often unevenly distributed. Those with greater socioeconomic disadvantage frequently benefit the least. This disparity is particularly pronounced in LMICs, including India, as substantial health inequalities exist and the uptake of interventions among the worse-off is limited.^14 15^ Extended cost-effectiveness analysis (ECEA) is used to evaluate the impact of a health policy instrument on equity-relevant outcomes.^16^ ECEA expands on standard cost-effectiveness analysis by including non-health benefits—like FRP alongside traditional health outcomes. ECEA allows for assessing how these benefits are distributed, which helps assess both affordability and equity.^16 17^

Previous economic evaluations of KMC from India have been based on studies with small sample sizes conducted in hospital settings.^18^ They have reported average cost-effectiveness, but the impact on FRP, that is, OOPE averted and catastrophic healthcare expenditure (CHE) averted, has not been reported. Also, they do not examine the distributional impact of the interventions geographically and across socioeconomic status.

In a large randomised controlled trial (RCT), we earlier showed that KMC initiated in a community setting does not increase survival inequity and may lower OOPE for households.^19 20^ In the current ECEA, we explore the cost and distributional consequences of scaling up KMC initiated in community settings through universal public financing. Specifically, we assess the impact on lives saved, disability-adjusted life-years (DALYs) averted and FRP provided to households overall and its distribution across geographical as well as socioeconomic status.

## Methods

### Target population

We conducted the analysis on a hypothetical cohort of all LBW newborns born in India in 2022. Data on the total number of live births and proportion born LBW were obtained from the United Nations population estimates and the National Family and Health Survey round 5 (NFHS-5).^7 21^ Given that 40% of all newborn deaths happen in the first 24 hours of life, our analysis cohort comprised 60% of all stable LBW newborns not requiring emergency care at birth.^22^

### Baseline coverage of interventions

KMC consists of skin-to-skin contact and exclusive breastfeeding. In the absence of direct KMC coverage data, we used the proportion of women receiving postnatal care with counselling on exclusive breastfeeding as a proxy measure, as these services are integral to KMC implementation. We used data from NFHS-5 to generate estimates for each socioeconomic quintile separately. The target coverage was set at 95% in the modelled scenario.

### Estimation of OOPE

Data on OOPE associated with care seeking among the enrolled infants were collected during follow-up visits at 28, 90 and 180 days of life. We included provider costs, medicines, diagnostics, transport and other direct medical/non-medical payments. Healthcare spending was collected for all morbidities and not restricted to LBW-attributable diagnoses. In the model, we applied arm-specific mean OOPE per inpatient episode and per outpatient visit, together with probabilities of inpatient-only, outpatient-only and combined care seeking, to estimate expected OOPE per infant by quintile.

Overall costs for care seeking by study arm (inpatient, outpatient and overall) have been presented earlier.^19^ The current analysis extends the earlier work by reporting the estimates for FRP at national level, overall and by quintiles and across different states in India.

### Model structure and outcomes

We used a decision tree model (online supplemental figure 1) to estimate the impact of scaling up KMC nationally to 95% coverage in India. Decision trees represent choices and probabilities leading to various outcomes. A decision node (square) represents a point where a choice between alternatives must be made. A chance node (circle) represents a point where possible outcomes have an associated probability, and the probability of all outcomes adds up to a terminal node (depicted as a triangle) which represents the end of a pathway. The associated outcomes in terms of health effects, costs or both are estimated at this stage, and the decision tree is completed. Further details are available elsewhere.^23^

Babies would enter the cohort if they were stable and survived the first 24 hours of life. They could either die before or survive until 28 days of life. Those who survived this neonatal period could either die during the postneonatal period or survive infancy. The same model was replicated for all socioeconomic quintiles using quintile-specific estimates.

Socioeconomic quintiles were based on the NFHS-5 household wealth index (asset based), and all NFHS-derived parameters (LBW prevalence, baseline coverage, household size, etc) were generated on asset index-based quintiles. Because CHE is defined relative to household income, we additionally generated quintile-specific household income distributions calibrated to national gross domestic product (GDP) per capita and the Gini coefficient for India. These income distributions were used only for the CHE calculations.

We generated the following estimates across wealth quintiles at national level and at state level in India, that is, geographically:

- Health outcomes
  1. Lives saved.
  2. DALYs averted.
- Non-health outcomes
  1. OOPE averted.
  2. CHE averted.
  3. Incremental cost-effectiveness ratio (ICER).

Since healthcare decision-making rests primarily with individual states in India, estimating these at state level is essential so that policymakers can determine which regions to focus on when expanding coverage.

Table 1 summarises the input parameters used in the model. Our RCT on KMC initiated in community settings was the primary source of data on effects, care-seeking patterns and costs of inpatient and outpatient care seeking.^19^ It was conducted among 8402 LBW newborns and showed a substantial reduction in the mortality of infants in the neonatal period and in the first 6 months of life.^9^ The recent systematic review on the effects of KMC, which included the data from our trial, reports similar estimates.^24^ All costs were reported in 2022 US$. The OOPE per episode increases with wealth, consistent with differential utilisation/provider choice and ability to pay. The cost estimates were adjusted for inflation between the year of data collection in the trial (2017–2018) and the year for which the model was developed, that is, 2022. Consumer price index available from the World Bank database was used for the adjustment. Cost and care-seeking data were based on RCT and were internally consistent. In addition to adjusting for inflation, we used sensitivity analysis to address the changes in prices or care-seeking patterns which would have occurred after the trial. To the best of our knowledge, updated estimates for healthcare-seeking costs among LBW infants were not available.

**Table 1:** Input parameters used in the model*

Parameter	Socioeconomic quintile					Source
	Lowest	Second	Middle	Fourth	Highest	
Proportion born low birth weight	0.21	0.20	0.17	0.17	0.15	NFHS^7^
Relative risk of death for KMC arm	0.67					Sivanandan and Sankar^24^
Mean OOPE for inpatient care seeking (current US$, 2018)—KMC arm	76.26	93.15	71.51	90.35	170.14	Analysis of primary data^19^
Mean OOPE for inpatient care seeking (current US$, 2018)—control arm	92.06	106.36	82.97	153.17	216.37	
Mean OOPE for outpatient care seeking (current US$, 2018)—KMC arm	9.40	11.82	9.75	8.74	10.44	
Mean OOPE for outpatient care seeking (current US$, 2018)—control arm	10.66	10.31	14.16	8.22	13.03	
Probability of inpatient care seeking	0.11					
Probability of outpatient care seeking	0.40					
Probability of inpatient and outpatient care seeking	0.09					
Baseline coverage of KMC	0.51	0.63	0.70	0.74	0.78	NFHS^7^
Target coverage level	0.95	0.95	0.95	0.95	0.95	Authors’ assumption
Proportion of women currently pregnant	0.047	0.039	0.036	0.036	0.031	NFHS^7^
Cost (current US$, 2022) of scale-up per low-birthweight newborn	91.1					WHO KMC Study^25^
Gini index for India	0.34					World Bank^26^
GDP per capita (current US$, 2022) for India	2411					World Bank^26^

* State-level parameters are shown in online supplemental table 1.

GDP, gross domestic product; KMC, kangaroo mother care; NFHS, National Family and Health Survey; OOPE, out-of-pocket expenditure.

The income distribution of the participating households represented the lower 50% of the income distribution in India. We used a gamma distribution to generate quintile-specific estimates of per capita income based on GDP per capita and the Gini coefficient for India.^16^ The GDP estimates were adjusted since only 60% of GDP for India is contributed by expenditure on personal consumption. We used the mean number of household members from NFHS-5 to convert per capita income into household-level estimates. We estimated neonatal and postneonatal mortality rates using the number of live births, reported deaths and age at death from the last 5 years in the NFHS-5.^7^

### Cost of scaling up KMC

The cost of scaling up the intervention in India was based on published literature.^25^ We used the reported incremental recurrent costs (of health worker time, supplies and operations) and start-up costs for KMC scale-up. The costs were estimated per live newborn eligible for KMC. Recurrent costs comprised staff time (full time equivaentsFTEs) for referral, counselling and discharge preparedness, provision and support of KMC by nurses and physicians, community-based follow-up after discharge, and programme management/supervision and recurrent training. Supplies and operational inputs included KMC wraps/garments, routine recording and educational materials, and food for mothers where provided, as well as transport and communication required for programme delivery. Start-up costs included establishment or renovation of KMC unit space (including toilets), furniture and basic amenities, development of communication materials and initial training. Further detailed information is available elsewhere.^25^

### Estimation of impact on DALYs averted

We estimated DALYs averted by converting deaths averted into years of life lost (YLL) averted. We did not model years lived with disability as mortality is the major driver of DALYs during the first year of life. First, we calculated lives saved among LBW infants as the product of (1) the incremental increase in KMC coverage under scale-up (target coverage minus baseline coverage), (2) the relative mortality effect of KMC from the published meta-analysis, (3) the number of LBW births eligible to benefit (live births by wealth quintile×LBW prevalence×an affected fraction to exclude deaths occurring before the intervention could plausibly act) and (4) quintile-specific mortality risk over the analytical mortality window (day 1 to day 180), expressed per 1000 live births. We then computed DALYs averted as YLL averted by multiplying lives saved by a life expectancy of 70 years.^26^

### Time horizon and discounting

We conducted the analysis for a time horizon of 1 year. Hence, we did not discount lives saved, OOPE averted, cases of CHE averted and cost of scale-up. Since DALYs averted would accrue over the lifetime of the infant, we discounted it at the rate of 3% per year consistent with standard economic evaluation practice.

### Statistical analysis

Data on OOPE overall and by quintiles at the national level were generated by multiplying the difference in OOPEs for KMC compared with routine care by the number of LBW children in each quintile based on the trial data. Catastrophic health expenditure (CHE) was modelled because direct household income/consumption data were not available at national scale for the target population. We therefore adopted a distributional modelling approach to estimate the probability that OOPE associated with care seeking for LBW infants would exceed a fixed share of annual household income/consumption. Annual household income was generated from a synthetic national income distribution parameterised using India’s GDP per capita and the national Gini coefficient; mean OOPE for LBW-related inpatient and outpatient care was taken from the community initiated Kangaroo Mother Care (ciKMC) trial, inflated to 2022 US$ and extrapolated from the trial follow-up period to an annual value. CHE cases were then calculated as the proportion of households in each quintile whose healthcare-related OOPE was greater than the CHE threshold of 10%. CHE averted under universal public financing and KMC scale-up was estimated as the difference in expected CHE cases between baseline coverage and the scale-up scenario, stratified by socioeconomic quintile and geography.

ICER was estimated based on the cost and effects for KMC in the scenario of scale-up compared with the existing level of coverage as follows:


$$\begin{array}{l} \left(Cost\;KMC\;scale-up\;–Cost\;KMC\;current\right)\\ /(Effect\;KMC\;scale-up\;–Effect\;KMC\;current). \end{array}$$

We adhered to the Consolidated Health Economic Evaluation Reporting Standards (CHEERS) guidelines.^27^ All analyses were done in R statistical environment V.4.4 and above.^28^

### Sensitivity analysis

We conducted one-way sensitivity analysis to identify the parameters with the highest impact on the ICER. SEs were applied to each parameter to assess uncertainty; where SEs were missing, we estimated them as ±25% of the mean. This approach helped us pinpoint specific variables that contributed most significantly to the variation in ICER, highlighting which factors most influenced the cost-effectiveness of the intervention including the estimates for cost and care seeking. We also created tornado plots based on the uncertainty ranges of different input parameters.

To account for simultaneous variations in multiple parameters, we performed probabilistic sensitivity analysis (PSA) using a Monte Carlo simulation with 100 000 iterations. For this analysis, we used beta distributions for probabilities, gamma distributions for costs and lognormal distributions for effect sizes. The beta distribution is suitable for probability values as it spans from 0 to 1, capturing a realistic range of outcomes. The gamma distribution is ideal for cost data as it models values starting from 0 and extending upwards without limit, accommodating widely varying costs. The lognormal distribution was used for effect sizes, which are positive and may be right skewed, with most values close to 1 and a few larger values representing stronger effects. We employed a triangular distribution for discounting using only a minimum, maximum and most likely (mode) value, rather than a full set of data points.

These distributions allowed the analysis to simulate real-life variations in both probabilities and costs, providing a more robust and realistic view of ICER under uncertain conditions, overall and by quintiles. The PSA results were illustrated on a cost-effectiveness plane, with median ICER values reported along with 2.5th and 97.5th percentiles.

### Ethics approval

The current ECEA was a modelling study based on data from multiple sources including those available in the public domain.

### Patient and public involvement

As this was a modelling study, patients or the public were not involved in the design, or conduct, or reporting, or dissemination plans of our research.

## Results

**Figure 1:**
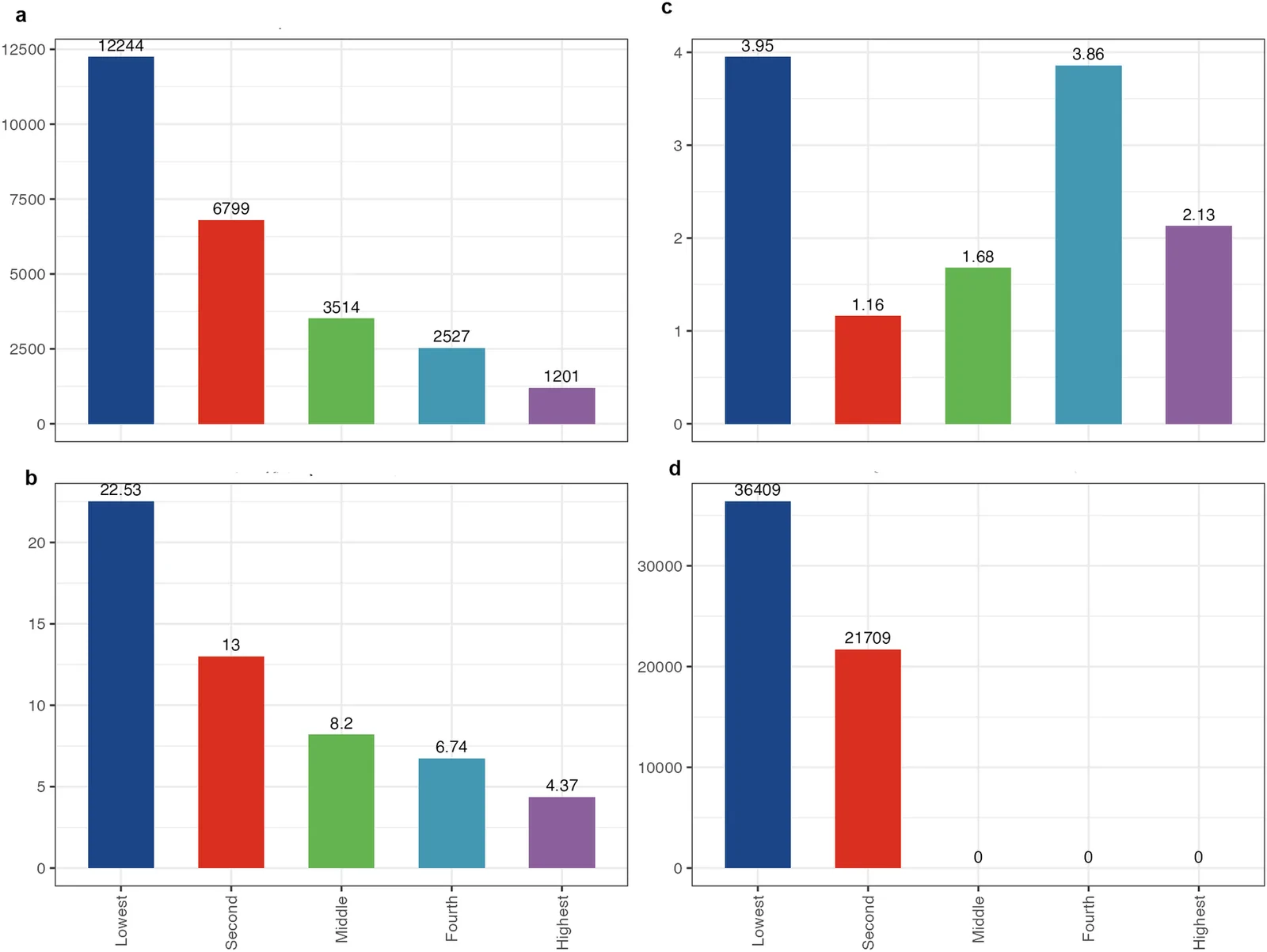
Impact of national scale-up of kangaroo mother care (KMC) using universal public finances per year on (a) lives saved, (**b**) cost of scale-up (million US$), (**c**) out-of-pocket expenditure (OOPE) averted (million US$) and (d) catastrophic health expenditure (CHE) cases averted.

Figure 1 presents the projected impact of nationwide KMC scale-up funded by public finances, including lives saved, OOPE averted, CHE cases prevented and total costs. Nationwide KMC scale-up is projected to save 26 285 lives per year, with approximately three-quarters of these benefits concentrated in the lowest two socioeconomic quintiles. It would also avert nearly US$13 million in out-of-pocket expenses and prevent over 57 000 households from experiencing CHE per year. The total cost of nationwide scale-up was estimated at US$55 million.

**Figure 2:**
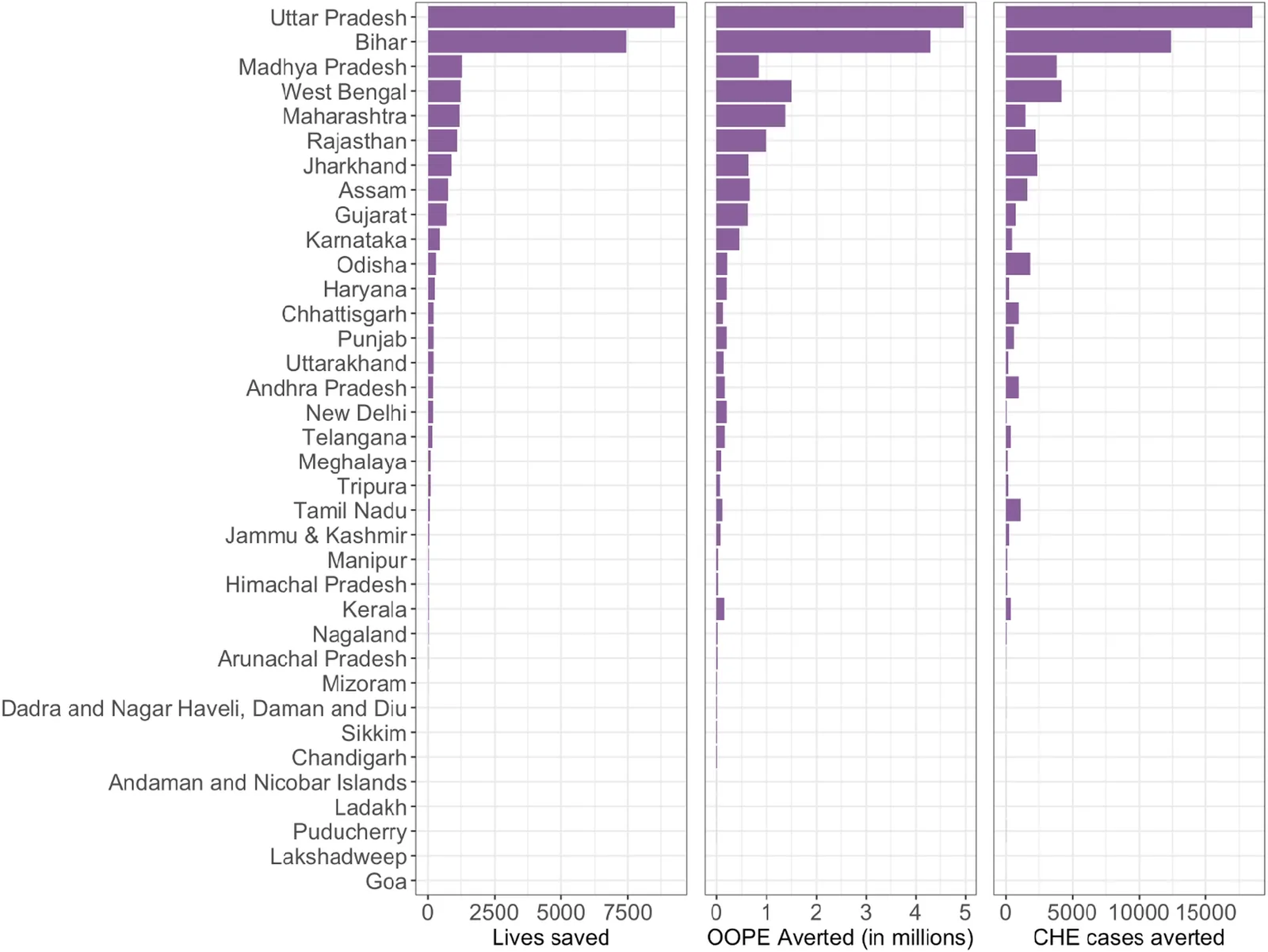
Impact of scale-up of kangaroo mother care (KMC) using universal public finance on infant lives saved, out-of-pocket expenditure (OOPE) averted and catastrophic health expenditure (CHE) cases averted across different Indian states.

Figure 2 shows that six states—Uttar Pradesh, Bihar, Madhya Pradesh, West Bengal, Maharashtra and Rajasthan—would account for over 90% of the benefits across all outcomes (lives saved, OOPE averted and CHE cases prevented).

Table 2 presents cost-effectiveness estimates by wealth quintile, with ICERs ranging from US$208 to US$412 per DALY. The greatest overall benefits—in terms of lives saved and CHE prevention—are projected for the lowest two quintiles, while the upper three quintiles would experience the largest reductions in OOPE.

**Table 2:** DALYs averted, cost of scale-up, ICER of scaling up kangaroo mother care and return on investment per million dollars invested in terms of lives saved, OOPE averted and CHE cases averted across different wealth quintiles per year

Outcome	Lowest	Second	Middle	Fourth	Highest	Total
DALYs averted	108 247	60 107	31 069	22 341	10 620	232 384
Cost of scale-up in US$	22 526 387	13 001 577	8 199 704	6 738 590	4 373 293	54 839 551
ICER (US$/DALY averted)	208	216	264	302	412	236
Lives saved per million	544	523	429	375	275	480
OOPE averted per million US$	175 144	89 086	205 220	572 601	487 670	223 169
Cases of CHE at 10% cut-off averted per million US$	1616	1621	0	0	0	1049

CHE, catastrophic health expenditure; DALYs, disability-adjusted life-years; ICER, incremental cost-effectiveness ratio; OOPE, out-of-pocket expenditure.

**Figure 3:**
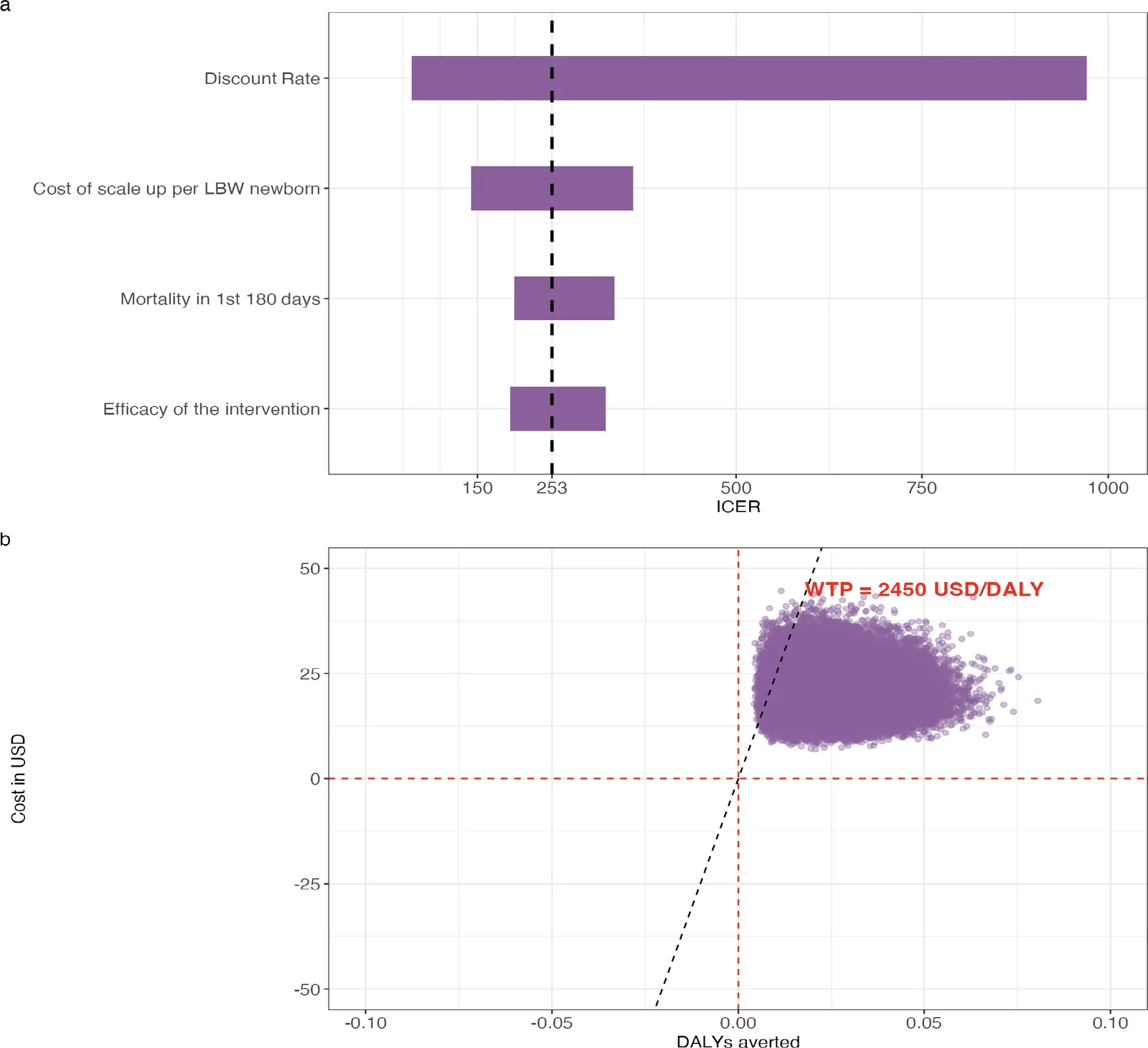
Sensitivity analysis (a) deterministic and (b) probabilistic for ICER. DALY, disability-adjusted life-year; ICER, incremental cost-effectiveness ratio; LBW, low birth weight; USD, US dollar; WTP, willingness to pay threshold.

Figure 3 presents deterministic sensitivity analysis and PSA. The discount rate had the largest impact on ICER, followed by the cost of scale-up per LBW newborn. KMC efficacy and mortality rates in the first 180 days had minimal influence. The PSA demonstrated that KMC scale-up remained cost-effective in 97.8% of simulations (using a willingness-to-pay threshold of 1× GDP per capita), ranging from 99.3% in the lowest quintile to 85.7% in the highest quintile. The median ICER at the national level was US$894 per DALY (2.5th, 97.5th percentiles: 387, 2361).

## Discussion

This study evaluates the health and financial impact of scaling up KMC in India using the ECEA framework. Our findings show that nationwide KMC scale-up could save approximately 26 000 lives, avert nearly US$13 million in OOPE and prevent an estimated 57 000 households from experiencing CHE per year. Importantly, three-fourths of these benefits would accrue to the lowest two socioeconomic groups. State-level analysis reveals that approximately 90% of benefits are concentrated in the six states with the poorest coverage, welfare and health outcome indicators, highlighting the equity-promoting nature of this intervention. Our findings remain robust across different assumptions, with both deterministic sensitivity analysis and PSA suggesting that scale-up of KMC is highly cost-effective under various conditions.

Scaling up KMC promotes equity across socioeconomic groups and geographical regions. Most of the benefits are concentrated among the most disadvantaged groups, both economically and geographically. The states poised to benefit most—characterised by lower GDP per capita, higher burdens of LBW and fragile health systems—also experience a disproportionately large share of neonatal deaths in India.^7 29 30^ By addressing these systemic inequities, scaling up KMC could deliver substantial health and financial gains to vulnerable populations, aligning with principles of distributive justice in healthcare.^6^

This study introduces methodological advancements to the ECEA framework, enhancing its robustness and policy relevance. Unlike traditional ECEAs, which primarily focus on socioeconomic quintiles and rely on secondary data, we incorporate geographical disaggregation and use primary data from a rigorously conducted RCT. This innovation addresses the decentralised nature of India’s health system, with substantial variations in healthcare quality and coverage between states. Geographical disaggregation enables a tailored approach to resource allocation, identifying high-gain states for targeted interventions. By leveraging RCT data, our analysis uses high-quality, evidence-based estimates for key parameters, including intervention effects, care-seeking behaviours and associated costs. This strengthens confidence in the findings and demonstrates how ECEA can complement classical RCTs to address equity and financial protection.

Our findings are consistent with earlier ECEAs evaluating health interventions in LMICs. Mao *et al* demonstrated substantial health and financial benefits from scaling up maternal and child health interventions in Nigeria using universal public finance, particularly for the poorest quintiles.^31^ Similarly, Nandi *et al* found that scaling up a home-based neonatal care package in India would substantially prevent neonatal deaths and reduce financial hardships.^32^ They estimated that scale-up to 90% would prevent six neonatal deaths and save US$4411 per 1000 live births in rural areas of India.

The annual cost of scale-up is at US$55 million. Although substantial, this represents a good investment. ICERs ranged from 0.08 to 0.17 times the current recommendation of the one-time GDP per capita willingness-to-pay threshold for India across all quintiles. The intervention was cost-effective for all except the topmost quintile even when alternate thresholds were used.^33 34^ Our analysis used data from a rigorously conducted RCT. Quintile-specific estimates for the majority of the input parameters were available, and we used standard techniques for scaling up the findings to the national or state level. Sensitivity analyses further reinforced the robustness of these findings, with 97.8% of probabilistic simulations indicating cost-effectiveness. Deterministic analysis identified the discount rate and cost of scale-up as the most sensitive parameters, underscoring the importance of these inputs in influencing cost-effectiveness estimates.

The major limitation of our study was the lack of nationally representative baseline KMC coverage data.

Our estimates for DALY are conservative, and hence our conclusions should not change even if years lost to disability are accounted for. We report the first ECEA of KMC from India with FRP outcomes (OOPE and CHE) and state-level geographical impact using primary RCT microcosting and care-seeking data. While the chosen proxy indicators may have led to underestimation of impact, the overall validity of our findings remains strong. Second, we assumed a uniform 95% scale-up coverage, which may not be feasible in all states due to variations in healthcare infrastructure and capacity; however, this assumption was justified to demonstrate the potential gains of scaling up the intervention. Future research should explore incorporating real-world implementation challenges into ECEAs. Additionally, our cost-effectiveness analysis used a 1-year time horizon, precluding consideration of long-term benefits such as improved cognitive outcomes and reduced non-communicable disease risks.^35^ While beyond the scope of this analysis, these represent important areas for future investigation.

## Conclusion

Scaling up KMC in India through universal public finance is a cost-effective and equity-enhancing intervention offering substantial benefits for health and FRP. We also demonstrate how the ECEA framework can enhance the utility of classical RCTs, provide new dimensions for evidence-based policy making and contextualise RCT findings. By targeting the poorest quintiles and high-burden states—which account for >90% of the benefits—this intervention has the potential to address critical health inequities in neonatal and infant mortality, aligning with India’s national health policy.

## Supplementary material

10.1136/bmjgh-2025-021719 online supplemental figure 1

10.1136/bmjgh-2025-021719 online supplemental table 1

10.1136/bmjgh-2025-021719 online supplemental file 1

10.1136/bmjgh-2025-021719 online supplemental file 2

## Data Availability

Data are available upon reasonable request.
