# Supplementary material for: Lives saved and financial risk protectionfrom scaling up kangaroo mother care in India: an extended cost -effectivenessanalysis
Source: BMJ Glob Health. 2026 Sep 2;11(9):e021719. doi: 10.1136/bmjgh-2025-021719 (PMC13548219; doi:10.1136/bmjgh-2025-021719)
Supplement: Supplementary file 1 [file bmjgh-11-9-s002.docx]

Supplement

Supplementary table 1. State level parameters used in the model.

| State | Population | GDP per capita (INR) | Birth rate | Currently Pregnant | Low birthweight |
| --- | --- | --- | --- | --- | --- |
| Andaman and Nicobar Islands | 402000 | 229080 | 10·6 | 1·9 | 17·4 |
| Andhra Pradesh | 52972000 | 197214 | 13·8 | 3·0 | 16·2 |
| Arunachal Pradesh | 1548000 | 204023 | 16·0 | 4·2 | 10·6 |
| Assam | 35378000 | 103964 | 16·8 | 4·1 | 16·1 |
| Bihar | 124919000 | 47450 | 25·9 | 6·2 | 16·8 |
| Chandigarh | 1219000 | 337538 | 11·7 | 2·3 | 16·7 |
| Chhattisgarh | 29836000 | 122934 | 16·4 | 3·7 | 15·9 |
| Dadra and Nagar Haveli, Daman and Diu | 644174 | 229080 | 16·5 | 3·4 | 20·8 |
| Goa | 1567000 | 475401 | 10·3 | 2·1 | 14·0 |
| Gujarat | 70648000 | 241930 | 15·0 | 3·5 | 18·5 |
| Haryana | 29846000 | 264729 | 16·4 | 3·8 | 20·5 |
| Himachal Pradesh | 7431000 | 195795 | 12·7 | 2·8 | 15·8 |
| Jammu & Kashmir | 13505000 | 116530 | 13·1 | 5·8 | 10·7 |
| Jharkhand | 38969000 | 84006 | 20·2 | 4·2 | 15·6 |
| Karnataka | 67268000 | 266866 | 14·2 | 3·2 | 15·9 |
| Kerala | 35633000 | 234435 | 11·8 | 3·4 | 16·3 |
| Ladakh | 299000 | 116530 | 12·6 | 5·8 | 11·6 |
| Lakshadweep | 68000 | 229080 | 12·5 | 3·4 | 9·7 |
| Madhya Pradesh | 85548000 | 116689 | 17·2 | 3·9 | 20·5 |
| Maharashtra | 125411000 | 215233 | 13·8 | 3·2 | 20·0 |
| Manipur | 3194000 | 91556 | 17·4 | 4·6 | 7·2 |
| Meghalaya | 3318000 | 103335 | 24·2 | 6·2 | 11·7 |
| Mizoram | 1227000 | 198962 | 15·0 | 3·9 | 4·0 |
| Nagaland | 2213000 | 121664 | 15·4 | 5·5 | 4·7 |
| New Delhi | 20965000 | 376217 | 14·8 | 2·9 | 22·1 |
| Odisha | 44162000 | 126437 | 15·9 | 3·3 | 19·2 |
| Puducherry | 1362786 | 249764 | 12·2 | 3·7 | 13·7 |
| Punjab | 30535000 | 169636 | 13·3 | 2·6 | 22·4 |
| Rajasthan | 80153000 | 133960 | 18·7 | 3·8 | 17·7 |
| Sikkim | 683000 | 463509 | 10·2 | 1·7 | 9·8 |
| Tamil Nadu | 76631000 | 242253 | 13·7 | 2·7 | 17·0 |
| Telangana | 37907000 | 269161 | 15·3 | 3·1 | 13·9 |
| Tripura | 4109000 | 137472 | 14·5 | 3·5 | 19·7 |
| Uttar Pradesh | 233297000 | 73884 | 20·5 | 4·1 | 20·2 |
| Uttarakhand | 11518000 | 205246 | 16·7 | 3·4 | 17·7 |
| West Bengal | 98604000 | 121904 | 15·5 | 3·1 | 19·0 |
