# Supplementary material for: Lives saved and financial risk protectionfrom scaling up kangaroo mother care in India: an extended cost -effectivenessanalysis
Source: BMJ Glob Health. 2026 Sep 2;11(9):e021719. doi: 10.1136/bmjgh-2025-021719 (PMC13548219; doi:10.1136/bmjgh-2025-021719)
Supplement: Supplementary file 2 [file bmjgh-11-9-s003.docx]

Contributors

TSC and KAJ designed the study with input from all co-authors. TSC, KAJ, and OAH developed the methodology. Data curation was carried out by TSC and SM. TSC conducted the formal analysis, while KAJ and ST accessed and verified the underlying data. HS and NB secured funding for the study. The initial draft was written by TSC, HS, and KAJ. All authors contributed to the interpretation of results, critically revised the manuscript, and approved the final version. All authors had the opportunity to request access to the underlying data and accept responsibility for submitting the manuscript for publication.

Funding

A grant for undertaking the study and supporting TSC’s and HS’s time was received from the Norwegian Research Council (RCN) through its Centres of Excellence scheme and the University of Bergen to the Centre of Intervention Science in Maternal and Child Health (project no. 223269) and EquiFinance (project no. 316354). OFN’s work on this study was supported by a Centre of Excellence grant to the Bergen Centre for Ethics and Priority Setting (project no. 332673). The funder of the study had no role in study design, data collection, data analysis, data interpretation, or writing of the report. The corresponding author had full access to all the data in the study and takes responsibility for the integrity of the data and the accuracy of the data analysis.

Competing interests:

All authors have completed the ICMJE uniform disclosure form at <https://www.icmje.org/downloads/coi_disclosure.docx> and declare no competing interests.

Data sharing

The dataset pertaining to the results reported in the manuscript will be made available to others only for health and medical research, subject to constraints of the consent under which the data was collected. Data will be available beginning 12 months and ending 5 years after publication of this article. Requests for data should be made to Dr Tarun Shankar Choudhary (tarun.choudhary@sas.org.in). The requester should provide a methodologically sound secondary research proposal, approved by an independent review committee. The requester must declare conflicts of interest in relation to the requested dataset and their funding sources. The authors reserve the right to refuse sharing of data in the face of potential adversarial conflicts of interest. A Data Sharing Agreement that meets the data sharing requirements of the Society for Applied Studies (New Delhi, India) and Centre for International Health, University of Bergen (Norway) will be signed with the data requester.

Acknowledgement

We acknowledge the contribution and support of the mothers and families of participating children. We acknowledge the hard work of all the data collectors and study team. The Society for Applied Studies acknowledges the core support provided by the Department of Maternal, Newborn, Child and Adolescent Health, World Health Organization, Geneva (WHO Collaborating Centre IND‐158); the Centre for Intervention Science in Maternal and Child Health, Centre for International Health, University of Bergen (Norway).
