# Supplementary material for: Lives saved and financial risk protectionfrom scaling up kangaroo mother care in India: an extended cost -effectivenessanalysis
Source: BMJ Glob Health. 2026 Sep 2;11(9):e021719. doi: 10.1136/bmjgh-2025-021719 (PMC13548219; doi:10.1136/bmjgh-2025-021719)
Supplement: Supplementary file 3 [file bmjgh-11-9-s004.docx]

### BMJ Global Health Author Reflexivity Statement

Adapted from Morton, B., Vercueil, A., Masekela, R., Heinz, E., Reimer, L., Saleh, S., Kalinga, C., Seekles, M., Biccard, B., Chakaya, J., Abimbola, S., Obasi, A. and Oriyo, N. (2022), Consensus statement on measures to promote equitable authorship in the publication of research from international partnerships. Anaesthesia, 77: 264-276. <https://doi.org/10.1111/anae.15597>

| **Study conceptualisation** | |
| --- | --- |
| 1. How does this study address local research and policy priorities? | The study assess the distributional impact of scaling up Kangaroo Mother Care, a WHO endorsed intervention for improving survival and growth among low birth weight newborns in India on survival as well non health outcomes like financial risk protection.  More than 2 million children every year in India are born low birth weight as thus the scale up of KMC is high priority. |
| 2. How were local researchers involved in study design? | Local researchers were involved in the conceptualisation, data collection analysis as well as interpretation of the study findings |
| **Research management** | |
| 3. How has funding been used to support the local research team(s)? | The funding was used to support the PhD of a local research team member as well as organising meetings/workshops on methods for assessment of fincial risk protection embedded within RCTs. |
| **Data acquisition and analysis** | |
| 4. How are research staff who conducted data collection acknowledged? | Yes |
| 5. How have members of the research partnership been provided with access to study data? | The data ownership of the primary trial lies with the local insitituion in India which conducted the research |
| 6. How were data used to develop analytical skills within the partnership? | Through workshops and PhD training. |
| **Data interpretation** | |
| 7. How have research partners collaborated in interpreting study data? | The partners analysed and interpreted the data keeping in mind the ground realities of implementing the intervention in India. Local health system capacity has been focussed on. |
| **Drafting and revising for intellectual content** | |
| 8. How were research partners supported to develop writing skills? | The first draft was written jointly by both research groups and led by the PhD candidate from India. Subsequent drafts were reviewed critically by the entire authorship team comprising of members from both institutions. |
| 9. How will research products be shared to address local needs? | Through disseminaton of the study finding with key stakeholders. |
| **Authorship** | |
| 10. How is the leadership, contribution and ownership of this work by LMIC researchers recognised within the authorship? | The lead author on the manuscript is a LMIC researcher. Other members who contributed are also authors on the manuscript. |
| 11. How have early career researchers across the partnership been included within the authorship team? | The lead author is an early career researcher and this is part of his PhD project. |
| 12. How has gender balance been addressed within the authorship? | Fair representation of both genders has been ensured for researchers who met the authorship criteria. |
| **Training** | |
| 13. How has the project contributed to training of LMIC researchers? | PhD fellowship and meetings/workshops |
| **Infrastructure** | |
| 14. How has the project contributed to improvements in local infrastructure? | Not applicable |
| **Governance** | |
| 15. What safeguarding procedures were used to protect local study participants and researchers? | Not applicable as this is a modeeling study. The primary study was approved by the institutional ethics committee at the Indian institution. |
